# Optimizing the accession-level quantity of seeds to put into storage to minimize seed (gene)bank regeneration or re-collection

**DOI:** 10.1093/conphys/coaf011

**Published:** 2025-02-23

**Authors:** Fiona R Hay, Katherine J Baum (née Whitehouse), Olaniyi Oyatomi, Dustin Wolkis

**Affiliations:** Department of Agroecology, Aarhus University, Forsøgsvej 1, 4200 Slagelse, Denmark; Australian Grains Genebank, Agriculture Victoria Research Division, Department of Energy, Environment and Climate Action, Private Bag 260, Horsham, VIC, 3401, Australia; International Institute of Tropical Agriculture, PMB 5320, Oyo Road, Ibadan 200001, Nigeria; Department of Science and Conservation, National Tropical Botanical Garden, 3530 Papālina Road, Kalāheo, HI, USA; Seed Conservation Specialist Group, Species Survival Commission, International Union for Conservation of Nature, Rue Mauverney 28, Gland 1196, Switzerland

**Keywords:** Genebank management, regeneration, seed bank, seed conservation, seed multiplication, seed storage

## Abstract

Seed (gene)banking is an effective way to conserve cultivated and wild plant diversity. However, long-term funding is not always consistently sufficient, and there is a need to both strengthen the effectiveness of genebank operations and maximize cost efficiency. One way to control the cost of maintaining a germplasm collection is to optimize the quantity of seeds per accession that is placed into storage, depending on the expected length of time a seed lot will remain above the viability threshold, expected rates of use for distribution and viability testing and on the requirement to ensure a reserve. Here, we express this as an equation, which can be applied to cultivated species and adjusted to different scenarios, but also to inform decisions about use of accessions of wild species where the number of seeds available is limited, a common scenario for wild-species conservation seed banks. For many crop genebanks, given the expected longevity of seeds, it would be worthwhile to increase the number of seeds produced and processed for storage. This would also help to diminish the risk of genetic drift due to frequent cycles of regeneration but would have implications in terms of how accessions are regenerated, in particular, how many plants are used for regeneration and the size of storage facilities. The equation we present can also be rearranged and used to plan how to allocate seeds for testing and use when the number of seeds available is limited. This may have particular relevance for species conservation seed banks.

## Introduction

While seed (gene)banking is the most efficient and low-risk method of *ex situ* plant conservation (Guerrant *et al.*, 2004; Walsh *et al.*, 2024), long-term conservation of germplasm comes at a cost. With wild-species seed banks and agricultural seed genebanks mostly relying on public or competitive funding, resources are often limited. One of the most significant costs for seed genebanks is that of regeneration, whereby seeds of an accession are sown to increase the seed quantity (‘multiplication’) or to replace a seed lot that has reached the low-viability threshold (‘rejuvenation’). For example, the cost of regeneration among genebanks of the European Cooperative Program for Plant Genetic Resources was ~45% of the total cost of running the genebanks, with the most significant cost being that of labour (Horna, 2008). Regeneration is also costly in terms of the risk of genetic drift (Parzies *et al.*, 2000; van Hintum *et al.*, 2007). This is the loss of the genetic integrity of the original germplasm due to the process of regeneration, e.g. in response to environmental pressures or due to harvesting and handling procedures. For this reason and because it is often difficult to cultivate wild species, re-collection is preferred by wild-species seed banks (Thompson *et al.*, 1981), but this too has significant costs and logistical challenges, especially if the species is found in far-away and/or inaccessible locations (Pedrini *et al.*, 2020; Rodríguez-Zúñiga *et al.*, 2022).

The risk of genetic drift and overall maintenance costs of a seed collection can be reduced by minimizing the regeneration/re-collection requirement of each accession (Sackville Hamilton and Chorlton, 1997; FAO, 2014). There are a number of ways to reduce the regeneration/re-collection requirement. Firstly, the viability threshold could be reduced, e.g. from 85%, which is the standard followed by many genebanks (Hay *et al.*, 2021) and that recommended in the *Genebank Standards* (FAO, 2014), to ≤75%. However, when the viability of a seed lot with high initial viability reaches 85%, it is entering the phase of most rapid decline in percentage viability (Hay and Sershen, 2021), thus it is not advisable to have a viability threshold <85%. Secondly, the longevity of the seeds could be increased. Assuming the storage conditions are already in accordance with the *Genebank Standards* (FAO, 2014) and therefore, in theory, optimized to a practicable extent, the best way to extend the longevity of a seed lot is to maximize the initial quality when it is placed into storage (Probert *et al.*, 2007; Whitehouse *et al.*, 2015, 2018; Kameswara Rao *et al.*, 2017). Again, we can assume that this is a given here, though the reality is probably that considerable improvements in longevity may be achieved by examining species-specific regeneration protocols and post-harvest processing practises (Hay and Probert, 2011; Ellis, 2019; Whitehouse *et al.*, 2020 and references therein). Indeed, this is a focus of the *Seed Quality Management* Community of Practice of the CGIAR Genebanks Initiative.

Another way to maximize the cost efficiency of seed (gene)banking is to optimize the quantity of seeds that is put into storage, by ensuring the quantity is ‘just sufficient to provide enough [seeds] for use before viability drops below threshold’ (Sackville Hamilton and Chorlton, 1997). In this context, ‘use’ refers to the seeds required for distribution and for viability testing—at least until such a time when we can non-destructively know the viability status of a seed lot in storage (Fu *et al.*, 2015). If too few seeds are placed into storage, it is likely that the accession will need to be regenerated or re-collected before the seed lot reaches the viability threshold. Conversely, putting more seeds into storage incurs inflated costs by collecting/producing and storing seeds that are never used, though they could be redirected towards research on seed physiology, such as seed longevity (Hay and Probert, 2013; Whitehouse *et al.*, 2020).

In this paper, we present a seed quantity optimization framework mathematically, with and without predictions of seed longevity, and discuss where it might be most useful to improve collection management. We also describe, using three case studies, how it can be used by seed genebanks (case studies 1 and 2) and conservation seed banks (case study 3) or when otherwise conserving seeds of non-domesticated species, to determine the quantity of seeds required for storage, as well as the quantity available for future viability testing and distribution.

## An Equation to Optimize Seed Quantity

The optimum quantity of seeds to store in a seed (gene)bank (active or base collection) for any particular accession depends on the total quantity of seeds distributed before the viability threshold is reached, the number of seeds required to monitor viability over that same period and, for crop genebanks, the minimum quantity required for regeneration. In the case of crop genebanks, the usage will depend on whether the seeds are in the active or base collection, i.e. whether the seeds are sampled to distribute to end-users (active collection) or whether they are primarily intended for long-term conservation (base collection) (Hay and Sershen, 2021). In the case of the base collection, according to the *Genebank Standards*, ‘It would be difficult to fulfil the function of a base collection unless accession size is sufficient to enable the accession to be regenerated, to provide an adequate sample to at least one active collection without regeneration and to allow at least a few monitoring tests of viability’ (FAO/IPGRI, 1994). Therefore, it was recommended that crop genebanks store 1500–2000 viable seeds, with 1000 seeds considered an absolute minimum. More recently, this was modified to 1500 for self-fertilizing species and 3000 for out-crossing species, and the minimum quantity of seeds should be enough for ‘three sowings of a representative population of the accession’ (FAO, 2014). The quantity of seeds to store in the active collection is not specified, presumably in part because this is highly dependent on the crop (seed yield and volume), local facilities and rates of use.

For *ex situ* wild-species seed collections, the aim is to have representative allelic diversity for species reintroduction, *in situ* restoration and/or introduction into breeding programmes. Wild-species seed banks generally maintain a single collection, not an active and a base collection. The primary purpose of this single collection is long-term conservation, often under conditions that are equivalent to those used for the base collection in crop genebanks. Examples of institutes that only conserve seeds at −20°C include the Millennium Seed Bank of the Royal Botanic Gardens Kew (Royal Botanic Gardens Kew, 2022) and many other botanic gardens. Since the purpose differs, rates of usage and management practises, also differ, although the genebank standards (FAO, 2014) are often used as the basis for ‘best practise’. In terms of number of seeds to put into storage, the *Seed Conservation Standards for ‘MSBP Collections’*, under standard 3.4 has ‘Collection size is monitored to ensure that sufficient potentially viable seeds are available for effective management and distribution to users’ (Royal Botanic Gardens Kew, 2022). The meaning of ‘effective management’ is not elaborated. Citing the Center for Plant Conservation (2019) and Way (2003), Cochrane *et al.* (2021) recommend a collection size of 3000–20 000 seeds. This latter reference also presents an interesting case study by Crawford and Monks, discussing the number of seeds to store depending on the rate of conversion into reproductively mature plants.

For now, as not all genebanks have both an active and a base collection, we will focus on mathematically expressing an optimum quantity of seeds to store in a genebank active collection to ensure we factor in all seed allocation requirements (i.e. viability monitoring, regeneration/re-collection and distribution), thus:


(1) \begin{equation*} Q=\left[{n}_{\mathsf{vt}}\times \left(y\div vt\right)\right]+\left[{n}_{\mathsf{d}}\times \left(y\times x\right)\right]+{q}_{\mathsf{min}} \end{equation*}

**Figure 1:**
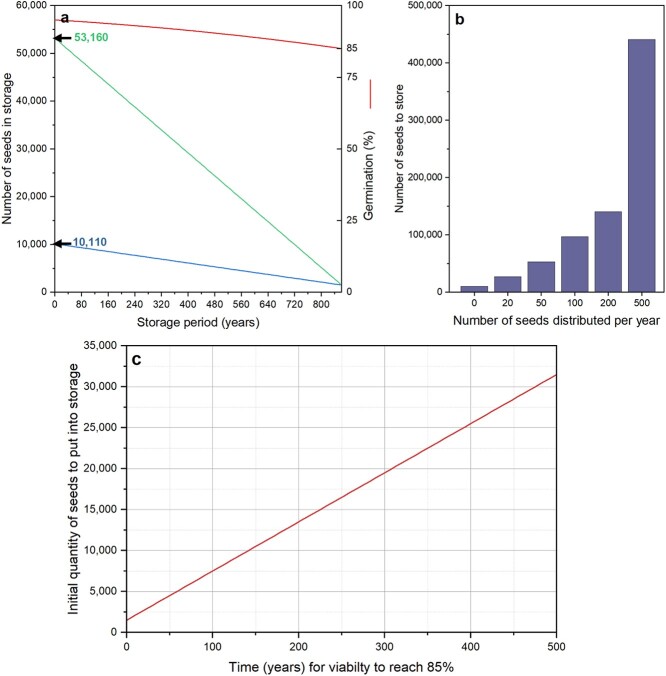
Theoretical number of seeds to store (*Q*) according to Equation 1. (**a**) Calculation for a *V. radiata* seed lot with 95% initial viability. The viability is predicted to reach the viability threshold of 85% during hermetic storage at 5°C after drying to equilibrium with 15% RH at 15°C (6.2% moisture content) after 861 years. The optimum quantity of seeds to store is calculated according to Equation 1, with 100 seeds (*n*_vt_ = 100) per 10-yearly viability test (*vt* = 10), a *q*_min_ of 1500 and no distribution (*x* = 0; *y*-intercept at 10 110) or with one sample (*x* = 1) of 50 seeds (*n*_d_ = 50) distributed each year (*y*-intercept at 53 160). (**b**) Number of seeds to store depending on the number of seeds distributed per year, for the same *V. radiata* seed lot. (**C**) Initial quantity of seeds to store depending on time for viability to fall to 85%, with 100 seeds used for viability testing (*n*_vt_ = 100) every 10 years (*vt* = 10) and 50 seeds distributed each year (*x* = 1, *n*_d_ = 50). For all these examples, the reserve quantity, *q*_min_, is set at 1500 seeds.

In Equation 1, the quantity (number of seeds) to store, *Q*, depends on the predicted longevity, *y* (years for viability to fall to a threshold value); number of seeds used for a viability test, *n*_vt_ or for distribution, *n*_d_; the viability test interval, *vt* (years); the expected number of samples distributed per year, x; and the minimum quantity of seeds that should be conserved (i.e. for regeneration), *q*_min_. The two terms in square brackets in the right of this equation both include the predicted seed longevity, *y*. This can be done for some species (those species with known viability constants)—in theory at the level of each individual seed lot—based on the initial viability test result and using the seed viability equations (Ellis and Roberts, 1980; Ellis, 2022). Indeed, the viability equation could be formulated and inserted into Equation 1 to substitute for *y*:


(2) \begin{align*} Q&=\left[{n}_{\mathsf{vt}}\times \left(\left[{\left({K}_{\mathrm{i}}-1.036\right)\times \sigma }\!\left/ {365}\right.\right]\div vt\right)\right]\nonumber\\&+\left[{n}_{\mathsf{d}}\times \left(\left[{\left({K}_{\mathrm{i}}-1.036\right)\times \sigma }\!\left/ {365}\right.\right]\times x\right)\right]+{q}_{\mathsf{min}}, \end{align*}

where *K*_i_ is the initial viability in probits and *σ* is the time, in days, that it takes for viability to fall by 1 probit. This value, *σ*, depends on the moisture content and temperature of the seeds in hermetic storage, based on parameters that are determined for each species. For example, if a seed lot of *Vigna radiata* with 95% initial viability and 6.2% moisture content is stored in a genebank cold room at 5°C, the predicted time for viability to fall to 85% is 861 years (SER/INSR/RBGK, 2023). This would mean that if 100 seeds are used for viability monitoring (${n}_{\mathsf{vt}}$) every 10 years ($vt$) and with a reserve quantity of 1500 (${q}_{\mathsf{min}}$), the quantity of seeds to be stored, *Q*, would be 10 110 before any distribution (Fig. 1a). The quantity increases dramatically with increasing rates of requests: with just one request per year and the number of seeds distributed each time, *n*_d_, equal to 50, *Q* increases to 53 160 (Fig. 1a and b).

Rather than setting the viability monitoring interval as a set period, such as 10 years, an alternative is to set ‘viability monitoring test intervals at one-third the time predicted for viability to fall to the determined regeneration threshold, but not exceeding 40 years’ (FAO, 2022). In other words, *vt* is set as *y*/3, and the number of viability tests is three. This simplifies Equation 1 to:


(3) \begin{equation*} Q=\left[{n}_{\mathsf{vt}}\times 3\right]+\left[{n}_{\mathsf{d}}\times \left(y\times x\right)\right]+{q}_{\mathsf{min}} \end{equation*}

and the optimum quantity to store then varies depending on the rate of use of seeds for distribution rather than for viability testing.

The most significant parameter in these equations is the expected longevity of the seeds (Fig. 1c), which, as indicated above, can be predicted using the viability equations. In reality, however, predictions of longevity based on the viability equations should only be used as an indicator of longevity; we now know that there is more variation in the longevity of different genotypes of a species stored under identical conditions than originally supposed (Whitehouse *et al.*, 2018; Lee *et al.*, 2019). Furthermore, the predictions for many crop species are so long that, due to the length of time we have been storing seeds in genebanks, we do not know their reliability. Thus, it is far more likely that *y* will be estimated based on the past performance of seed lots in genebank storage (e.g. Yamasaki *et al.*, 2020; Hay *et al.*, 2021). For some species, with particularly long-lived seeds, there is not yet sufficient data to set an informed value for *y* and hence, it may be necessary to choose a reasonable but nonetheless ‘safe’ value, perhaps the period that the majority (95% perhaps) of seed lots maintain viability above the threshold. This requires examination of the overall performance of seed lots harvested in particular years (Agacka *et al.*, 2013, 2014; Hay *et al.*, 2015).

It is important that the genetic potential of the material stored in genebanks is realized, but this can only happen when it is distributed and used by breeders, restorationists and/or other scientists. High rates of distribution are important to justify the long-term conservation and maintenance of plant genetic resources (Halewood *et al.*, 2020). Optimizing seed quantity for storage would require genebanks to predict the ‘expected’ usage for each accession. This is generally very difficult to achieve unless, for example, a genebank has been contracted to have available a specific set of material due to a request from industry. Even then, there is an element of uncertainty in relation to future request rates. Further, it may well be that particular accessions fall in and out of favour in terms of requests, due to changes in breeding, research and/or conservation priorities. One approach that might be helpful is to use the historical records of requests. These data are usually included in the Genebank Information Management System (Galluzzi *et al.*, 2016). Indeed, Equation 1 could be set to work in the background of the system to calculate *Q* based on both historical distribution data and viability data (to inform longevity) for the accession. When a sample is identified for regeneration, the system could estimate an optimum seed quantity that can then be used to calculate the number of plants required for regeneration and/or how many seeds to harvest and process for storage.

## Case Study 1

### Optimizing seed quantity for *Triticum aestivum* at the Australian Grains Genebank

**Figure 2:**
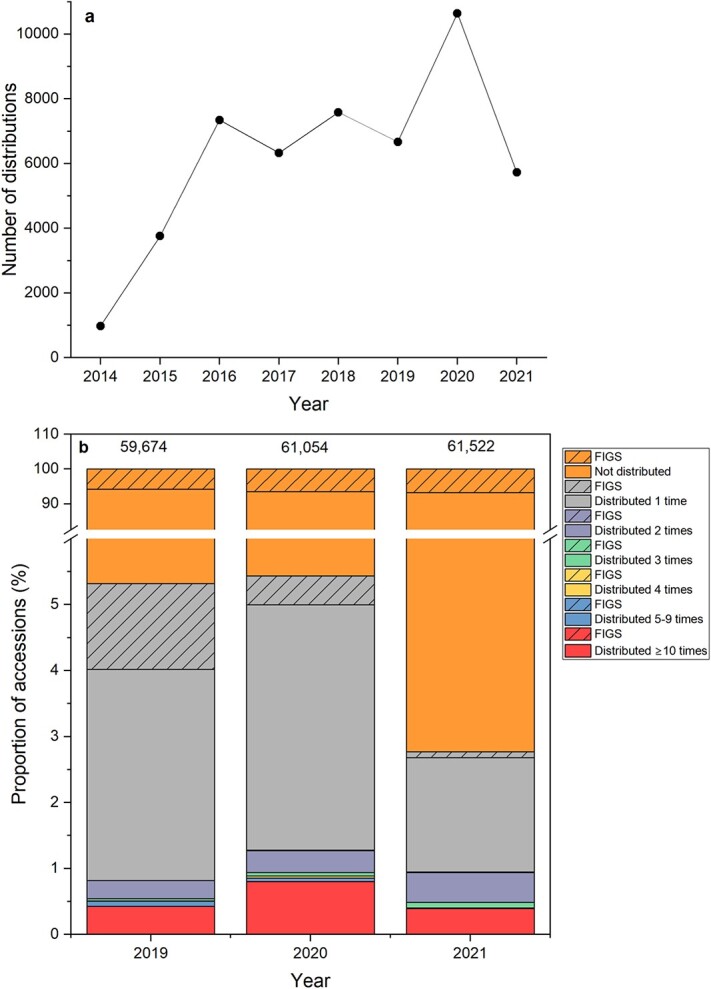
(**a**) Number of *T. aestivum* samples distributed since 2014 by the AGG and (**b**) proportion of accessions, including those within a FIGS core set, that were distributed between 2019 and 2021, at different frequencies. The number at the top of each column in (**b)** is the total number of accessions.

Wheat (*Triticum aestivum*) is one of the most accessed crops at the Australian Grains Genebank (AGG). Samples are distributed for use in both public and private pre-breeding and breeding programmes, with 95% of distribution within Australia and 5% international. Seeds at the AGG are dried at 15°C and 15% relative humidity (RH) prior to storage at −20°C. Under such conditions, *T. aestivum* seeds are typically long-lived with a predicted longevity (time for viability to fall by 1 probit) of 528 years estimated using the viability constants available in the Seed Information Database (SER/INSR/RBGK, 2023). The initial viability of *T. aestivum* accessions at the AGG is typically high, averaging 90.4%. Since 2014, when the AGG was established and germplasm moved to the new facility in Horsham, the number of *T. aestivum* samples distributed annually has increased from 977 in 2014 to a peak of 10 646 in 2020 (Fig. 2a). This significant increase from previous years was due to an increased focus on querying and requesting germplasm by researchers and breeders who had changed working conditions during the early stages of the COVID-19 pandemic. In 2021, 5725 samples were distributed (between January and September), with levels in line with pre-pandemic levels. As of 2022, there were 62 271 accessions, many of which have never been distributed since they were deposited into the AGG or precursor genebanks (over 60 years). More recent acquisitions by the AGG are aligned to active research and breeding programmes, with a distribution of nearly all accessions at least once since deposited. For example, 356 of the 376 *T. aestivum* accessioned into the AGG in 2014 have been distributed at least once.

In 2019 and 2020, 5.3 and 5.4% of *T. aestivum* accessions, respectively, were distributed at least once; in 2021, the proportion was 2.8% (Fig. 2b). Some accessions are requested very frequently; between 0.4 and 0.8% of accessions were distributed at least 10 times between 2019 and 2021. Core sets, e.g. sets of accessions selected by following a focused identification of germplasm strategy (FIGS; Khazaei *et al.*, 2013), are often accessions with higher rates of distribution, at least over an initial period when the basis for the FIGS is most relevant in relation to breeding targets.

Currently, the AGG aims to produce ~2800 seeds per accession in order to bank a minimum number of 2000 seeds. This buffer ensures there are enough seeds to carry out an initial viability test prior to storage (100 seeds) as well as for safety duplication at both the National backup facility (200 seeds) and the Svalbard Global Seed Vault (200 seeds). These samples are removed before the remaining bulk of seeds is packed and placed into storage. Therefore, the 2000 seeds stored per accession is the active sample used for distribution, periodic viability monitoring and regeneration. Typically, for *T. aestivum* accessions, the number of seeds distributed per request is 100 and viability monitoring is tested every 10–20 years. When applying these quantities to Equation 1, it is clear that a 2000-seed minimum is a drastic under-estimation given the predicted longevity of *T. aestivum* seeds; especially when the initial viability is quite high (Table 1). For example, when storing seeds at 6.1% moisture content and at 4°C, the quantity of seeds to be stored for an accession showing an initial viability of 90% would be 2160 before any distribution. This quantity increases to 2400 when initial viability is 95%, and to 10 400 seeds when combined with a distribution rate of once per year (100 seeds per request). Note, when storing seeds under conditions that further promote seed longevity (e.g. under long-term storage conditions, −20°C), a greater quantity of seeds will be required for storage (Table 2).

**Table 1:** Optimized quantity of *T. aestivum* seeds to store in the active collection at the AGG based on predicted longevity ($y$) and number of samples distributed per year ($x$), with 100 seeds distributed per request (${n}_{\mathsf{d}}$), using Equation 1. No viability testing. No minimum quantity (${q}_{\mathsf{min}}$

Initial viability (%)	Predicted time for viability to fall to 85% (years)^a^	Number of samples distributed per year							
		0	1	2	3	4	5	10	20
99	169	2845	19 745	36 645	53 545	70 445	87 345	171 845	340 845
98	133	2665	15 965	29 265	42 565	55 865	69 165	135 665	268 665
97	111	2555	13 655	24 755	35 855	46 955	58 055	113 555	224 555
96	93	2465	11 765	21 065	30 365	39 665	48 965	95 465	188 465
95	80	2400	10 400	18 400	26 400	34 400	42 400	82 400	162 400
94	68	2340	9140	15 940	22 740	29 540	36 340	70 340	138 340
93	58	2290	8090	13 890	19 690	25 490	31 290	60 290	118 290
92	48	2240	7040	11 840	16 640	21 440	26 240	50 240	98 240
91	40	2200	6200	10 200	14 200	18 200	22 200	42 200	82 200
90	32	2160	5360	8560	11 760	14 960	18 160	34 160	66 160
89	25	2125	4625	7125	9625	12 125	14 625	27 125	52 125
88	18	2090	3890	5690	7490	9290	11 090	20 090	38 090
87	12	2060	3260	4460	5660	6860	8060	14 060	26 060
86	6	2030	2630	3230	3830	4430	5030	8030	14 030

a Calculated assuming seeds are dried at 15% RH and 15°C (predicted equilibrium moisture content 6.1%, assuming seeds have an oil content of 2.2%) and stored at 4°C (SER/INSR/RBGK, 2023).

**Table 2:** Quantity (Q) of seeds to store in the medium-term (MTS; 5°C) and long-term storage (LTS; −20°C) collections for the top 10 crop species held in the worlds’ genebanks (Wambugu *et al.*, 2018) and the wild species *Digitalis purpurea*. Quantities, before distribution (i.e. $x$ = 0) were estimated using Equation 1 and based on predicted longevities (SER/INSR/RBGK, 2023), with 100 seeds used for viability monitoring (${n}_{\mathsf{vt}}$) every 10 (MTS) or 20 years (LTS)($vt$) and with a reserve quantity of 1500 seeds (${q}_{\mathsf{min}}$)

Species	Predicted time for viability to fall from 95 to 85% in the MTS (years)^a^	Q	Predicted time for viability to fall from 95 to 85% in the LTS (years)^a^	Q
*Triticum aestivum*	80.6	2306	354.4	3272
*Oryza sativa*	63	2130	277.1	2886
*Hordeum vulgare*	107.8	2578	474.1	5871
*Zea mays*	81.9	2319	360.1	3301
*Phaseolus vulgaris*	269.6	4196	1185.6	7428
*Sorghum bicolor*	489.7	6397	3837.7	20 689
*Glycine max*	49.0	1990	215.3	2577
*Avena sativa* *				
*Arachis hypogaea*	30.8	1808	135.5	2178
*Gossypium hirsutum*	380.8	5308	2583.6	14 418
*Digitalis purpurea*	1.3	1501	5.9	1530

a Calculated assuming seeds are dried at 15% RH and 15°C (SER/INSR/RBGK, 2023).

b Viability constants have not been determined for *A. sativa*.

## Case Study 2

### Optimizing seed quantity for *Vigna subterranea* and *Sphenostylis stenocarpa* at the International Institute of Tropical Agriculture Genebank

Bambara groundnut (*Vigna subterranea*) and African yam bean (*Sphenostylis stenocarpa*) are two minor Legume crops that nonetheless play a significant role in food security in parts of Africa (Tan *et al.*, 2020; Adewale and Nnamani, 2022). Of the >3000 accession records of Bambara groundnut in Genesys (a global, open access portal for crop genetic resources conserved in genebanks around the world), two-thirds are managed by the genebank at International Institute of Tropical Agriculture (IITA) (https://www.genesys-pgr.org/a/v2PleWYb6X9; 12 September 2023). Similarly, of the >1300 accession Genesys records for African yam bean, ~40% are held at IITA (https://www.genesys-pgr.org/a/v23DRDGMkQg; 12 September 2023). Seeds of these species are known to be orthodox (SER/INSR/RBGK, 2023), however, the viability equation constants have not been determined for either of these two species; thus it is not possible to make meaningful predictions of their seed longevity. In an analysis of historical viability monitoring data for accessions of Bambara groundnut stored in the IITA genebank, the germination data (collated from germination tests using 50 seeds per seed lot) ranged from 0 to 100% for seeds tested after <12 years or after 22–27 years in medium-term storage (Hay *et al.*, 2021). This broad germination range suggests non-optimal dormancy-breaking/germination testing procedures, but also that seeds of some seed lots at least, have the capacity to maintain levels of viability greater than the 80–85% viability threshold (*y* in eqn. 1) for ≥25 years. In the absence of further evidence of longevity in genebank storage, a 10-year monitoring interval would be acceptable, i.e. *vt* (eqn. 1) = 10. The same 10-year interval could also be set for African yam bean, for which historical genebank monitoring data have not been collated. For each species, IITA distributes each accession on average once every other year, i.e. *x* (eqn. 1) = 0.5, with each distribution sample comprising 100 seeds, *n*_d_ = 100. Both species self-pollinate, giving a ${q}_{\mathsf{min}}$of 1500 seeds. Accordingly, we can calculate the optimum quantity of seeds to put into storage, *Q* as:


(Equation 1, with values) \begin{equation*} Q=\left[50\times \left(25\div 10\right)\right]+\left[100\times \left(25\times 0.5\right)\right]+1500 \end{equation*}

which gives 2875 seeds. This is very much higher than the current average quantity of 1000 seeds.

## Regeneration Protocols and Genebank Infrastructure

The equation suggests, based on the theoretical data presented here, that much larger quantities of seeds should be stored than is currently normal practise; especially when stored under long-term storage conditions (Table 2). For example, in the case of *V. radiata*, discussed above, the quantity of seeds to store is at least 10 110 (Fig. 1). With a thousand-seed weight of 31.8–68.4 g (SER/INSR/RBGK, 2023), the weight of seeds to put into storage would be in the region of 321.5–691.5 g. With even one sample of 50 seeds distributed per year, the weight of seeds to put into storage would be 1.7–3.6 kg. It is important to note however, that increasing the quantity of seeds in storage for each accession should not be achieved by increasing the number of seed lots. Having ‘multiple seed lots’ of an accession in the active or base collection reduces the management efficiency and increases costs, e.g. in relation to viability monitoring as each seed lot should be tested. Indeed, some of the CGIAR genebanks have been rationalizing their collections due to multiple seed lots, evaluating all the seed lots for each accession and only keeping those with good viability (ideally, ≥85%) and reasonable quantity (Hay *et al.*, 2021).

There are two important consequences of increasing seed quantity: (i) the plot size/number of plants that are used for regeneration and (ii) the space required to store such quantities of seeds. The number of plants to use in sampling and regeneration should be informed by the mating system (i.e. self- or cross-pollination), population size, migration and fecundity; and/or measures of genetic diversity (Brown and Marshall, 1995; Johnson *et al.*, 2002; Reyes-Valdés *et al.*, 2018; Hoban, 2019). Current best practise advises collecting from 50 individuals for self-pollinating species and from 25 individuals for cross-pollinating species per population (Crossa and Vencovsky, 2011), although many more should be sampled if the aim is to ensure that a target proportion of alleles (i.e. 95%) is captured at least once (Hoban, 2019). Different sampling strategies will yield different proportions of alleles (Hoban *et al.*, 2018) and the rate of capture of alleles, depending on how many plants and populations are sampled, varies among species (Griffith *et al.*, 2019). Guidance regarding how many plants to use for regeneration of *ex situ* collections is limited. According to the *Crop Genebank Knowledge Base* (https://cropgenebank.sgrp.cgiar.org/ accessed 9 June 2023), a compilation of standard operating procedures from various genebanks, the quantity of seeds used for regeneration is, e.g. 480 for chickpea; for rice, it is 100 transplanted seedlings; for cowpea, it is 80 seeds (or a minimum of 50); for radish, it is 300 seeds to have 100 plants for heterogenous accessions or 100 seeds for 30 plants for genetically fixed accessions. It may be necessary, depending on the species (and accession), to increase plot sizes and the number of plants to ensure sufficient quantities of seeds can be harvested. Alternatively, it may be possible to increase the planting density, although care must be taken to ensure that plant and seed development are not limited and to minimize disease and/or insect pressure.

Some seed (gene)banks have large cold storage rooms and may have some spare capacity to increase the quantity of seeds stored per accession or even dedicated space that is waiting to be commissioned. Others may be able to expand by acquiring more fridges/freezers. Adjusting quantities may be a process, and take some years to do, increasing the number of seeds for an accession only when regeneration is needed. If that need is driven by low quantity of seeds, this may be a way to prioritize accessions for increased seed quantity.

## What to Do when Seed Numbers Are Limited

It can be challenging to collect and/or produce enough seeds of some wild species (Way, 2003; Hay and Probert, 2013), including those that are rare, crop wild relatives and forages. For example, Way (2003) reported that of 1022 seed collections made by a seed collector from the Royal Botanic Gardens Kew, more than half were estimated to have <10 000 seeds and 10% were estimated to have <1000 seeds. With very small collections, especially those with high conservation value (i.e. species that are extremely rare, endangered and/or extinct in the wild), the decision may be taken not to test viability at all, especially if dormancy-breaking/germination requirements are unknown. While more and more dormancy and germination data are being collected and compiled (e.g. Fernández-Pascual *et al.*, 2023; SER/INSR/RBGK, 2023), for some wild species, it might still take a number of laboratory trials before germination and/or viability results are considered acceptable (Hoyle *et al.*, 2023).

**Figure 3:**
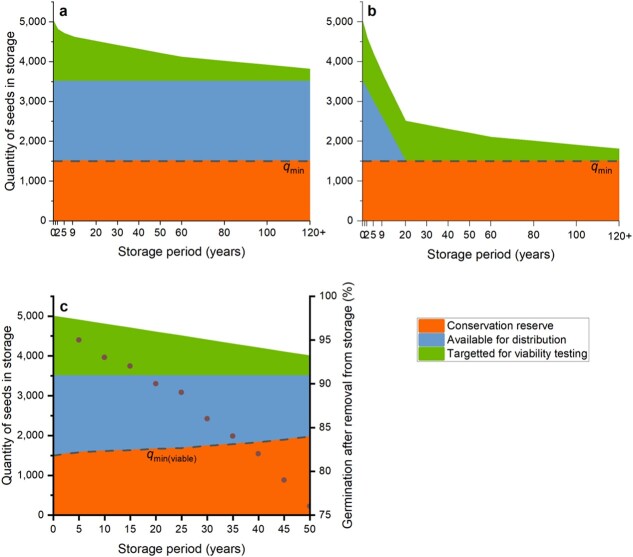
Various scenarios for allocating seeds when the quantity placed into storage is limited. (**a**, **b**) An inventory comprising 5000 seeds, with a minimum quantity, *q*_min_, set at 1500 seeds. In this example, 100 seeds are used for viability testing (*n*_vt_ = 100) after each of 1, 2, 5, 9, 20, 30, 40, 50, 60, 80 and 100 years, and assuming viability is maintained above the viability threshold; three further samples are available for viability testing. In (A), no seeds are distributed (*x* = 0); in (B), 100 seeds are distributed each year (e.g. *x* = 1, *n*_d_ = 100 or *x* = 2, *n*_d_ = 50). (**c**) An inventory comprising 5000 seeds, with a minimum quantity, *q*_min_, set at 1500 viable seeds; 100 seeds are used for viability testing (*n*_vt_ = 100) every 5 years (*vt* = 5) and *q*_min_ is adjusted according to the viability test result (symbols—not real data; usually determined by a germination test).

For accessions with a limited number of seeds, it might be more useful to express Equation 1 as how many of the seeds that are stored should be used for viability testing and/or distribution, i.e.:


(4) \begin{equation*} \left[{n}_{\mathsf{vt}}\times \left(y\div vt\right)\right]+\left[{n}_{\mathsf{d}}\times \left(y\times x\right)\right]=Q-{q}_{\mathsf{min}} \end{equation*}

The number of seeds made available for distribution may vary, even during storage, depending on internal or external mandates and priorities, and the conditions under which the seeds were acquired (Fig. 3). In general, rates of distribution by species conservation seed banks tend to be low compared with crop genebanks, and regeneration may not be part of routine processes, with resources more likely to be used to re-collect a species if the viability of the original accession declines below the threshold—in which case, *q*_min_ could even be set to zero. In using this version of the equation, given the nature of the species, it is likely that the longevity of the seeds is not known, although ranking may be possible based on taxonomy and/or origin, or by conducting seed storage experiments (Probert *et al.*, 2009; Mondoni *et al.*, 2011). Some desiccation-tolerant seeds are extremely short-lived, even under genebank storage conditions, while others are extremely long-lived (Chau *et al.*, 2019; Colville and Pritchard, 2019). Hence, for a species with no longevity data, it may be appropriate to use a monitoring schedule with shorter intervals at the beginning and increasingly longer intervals if viability stays high, as used in experimental comparative seed longevity testing (Newton *et al.*, 2014; Hay *et al.*, 2022). Alternatively, more conservative monitoring intervals, e.g. every 2–5 years might be set. The equation could also be modified such that a constant quantity of viable seeds—or seeds that are expected to be converted into mature plants—becomes the minimum quantity that should be conserved and not used for distribution:


(5) \begin{equation*} \left[{n}_{\mathsf{vt}}\times \left(y\div vt\right)\right]+\left[{n}_{\mathsf{d}}\times \left(y\times x\right)\right]=Q-\frac{q_{\mathsf{min}}}{via} \end{equation*}

where *via* is the proportion of viable seeds (Fig. 3c).

## Case Study 3

### Optimizing viability testing for *Cyanea kuhihewa* at the National Tropical Botanical Garden (USA)

Seed viability equation constants have not been determined for any native Hawaiian species, but a study of 295 species from the Hawaiian flora found that Hawaiian Campanulaceae seeds are desiccation-tolerant, but short-lived at conventional (−18°C) storage temperatures (Chau *et al.*, 2019). *Cyanea kuhihewa* Lammers. is a Kauaʻi Island (Hawaiʻi, USA) endemic plant species in the Campanulaceae family, assessed as ‘critically endangered’ on the International Union for the Conservation of Nature Red List of Threatened Species (Rønsted *et al.*, 2020). Originally discovered in 1991 with just one wild population of 12 individuals (Rønsted and Wood, 2020), the species declined to a single wild individual in 2019. National Tropical Botanical Garden (NTBG) Senior Research Biologist Ken Wood and collaborators from the Hawai’i State Department of Land and Natural Resources and The Nature Conservancy collected seeds from that individual in 2021. The individual died in 2023, though a new population comprising four individuals was discovered in late 2023 (K. Wood, NTBG, pers. comm.). At the time of writing, 2619 seeds from the 2021 collection (accession 20 210 378) are conserved in the NTBG Seed Bank and Laboratory.

 A congener of *C. kuhihewa*,*Cyanea kunthiana*, was shown to have non-deep simple morphophysiological dormancy (MPD), requiring >4 weeks for maximum germination to occur when incubated at 25/15°C (12-h light/12-h dark)(Baskin *et al.*, 2005, 2020). Three replicates of 15 *C. kuhihewa* seeds were each sown on Anchor germination paper in 60-mm-diameter Petri dishes to assess initial viability, which was found to be 73%. Although *C. kuhihewa* was not assessed specifically, based on 11 of 32 Hawaiian *Cyanea* species reported in Chau *et al.* (2019), we assume a re-collection threshold (when viability declines by 30% from initial/maximum viability) of 10 years, although, given that this individual is deceased, re-collection will not be possible, and regeneration should be pursued. Using Equation 4, we can determine how many seeds to allocate for future viability testing and ‘distribution’, perhaps to produce plants to supplement the extant population. Various scenarios are possible. For example, if most seeds are allocated to plant production, once per year (x = 1),


$$ \left[{n}_{\mathsf{d}}\times 10\right]=2619-{q}_{\mathsf{min}} $$

and if *q*_min_ = 0, the number of seeds to ‘distribute’ each time or per year, would be 262. If some seeds are kept as a reserve, e.g. *q*_min_ = 524 (20% of the original collection), the number of seeds to distribute per year would be 210. The number of seeds that develop into healthy plants could be used as a proxy for seed viability in the absence of viability testing. But, if viability testing is carried out every year, using 30 seeds each time, and again setting *q*_min_ = 0, we have


$$ \left[30\times \left(10\div 1\right)\right]+\left[{n}_{\mathsf{d}}\times 10\right]=2619 $$

which means that up to 232 seeds could be available for distribution per year for the 10 years. As above, if we put *q*_min_ = 524, up to 180 seeds could be available for distribution per year.

We can also use Equation 4 to calculate how many seeds would remain after a given storage period (e.g. 10 years, based on the re-collection threshold, above). For example, testing 20 seeds for viability every year, with 50 seeds distributed a year:


\begin{align*} Q\ after\ 10\ years&=2619-\left[20\times \left(10\div 1\right)\right]\nonumber\\&+\left[50\times \left(10\times 1\right)\right]=1919 \end{align*}

## Conclusions

Expressing an optimum quantity of seeds to put into storage based on expectations regarding seed lot longevity and rates of distribution suggests that seed (gene)banks should try to harvest or collect greater quantities of seeds than is currently normal. Doing so might be challenging in relation to the number of plants it is possible to harvest from and storage capacity. Nonetheless, we hope that in going through this exercise, seed (gene)banks might consider increasing the quantity of seeds placed into storage, albeit in all likelihood, not to the actual quantities suggested. As such, the equations given here and that could be incorporated into collection information management software and seed conservation protocols, can contribute to improved management of *ex situ* seed collections of both cultivated and wild plant species. Improving our understanding of the longevity of seed lots in storage through concerted efforts in conservation research will further improve the utility of the equations we have presented.

## Data Availability

No new data are associated with this work.
